# Non-Conjugated Linear Polysiloxane with Cluster-Triggered Circularly Polarized Luminescence

**DOI:** 10.1021/jacsau.5c01533

**Published:** 2026-01-13

**Authors:** Hao-Cheng Yu, Tomoki Mure, Towa Shinoda, Chi-Shan Lu, Kai Terami, Shunsuke Morii, Shih-Han Li, Tomoyasu Hirai, Ming-Chia Li

**Affiliations:** † Institute of Molecular Medicine and Bioengineering, 34914 National Yang Ming Chiao Tung University, Hsinchu 300, Taiwan; ‡ Center For Intelligent Drug Systems and Smart Bio-devices (IDS^2^B), National Yang Ming Chiao Tung University, Hsinchu 300, Taiwan; § Department of Biological Science and Technology, National Yang Ming Chiao Tung University, Hsinchu 30010, Taiwan; ∥ Department of Applied Chemistry, 57935 Osaka Institute of Technology, Osaka 535-8585, Japan

**Keywords:** circularly polarized luminescence, clusteroluminescence, polysiloxane, circular dichroism

## Abstract

In this study, we introduce a system that is both nonconjugated and nonaromatic, specifically engineered to generate luminescence via cluster-triggered emission in its aggregated state. By integrating a chiral moiety, *N*-(*tert*-butoxycarbonyl)-cysteine methyl ester (cys) as a side group into a linear poly­(methyl vinylsiloxane) (PMVS) backbone, we successfully achieved pronounced circularly polarized luminescence (CPL). The formation of the helical conformation was evaluated by vibrational circular dichroism (VCD) and electronic circular dichroism (ECD) spectroscopy. Furthermore, the 2D NMR analyses indicated that intramolecular hydrogen bonding significantly contributes to the stabilization of this structure, because of the intrinsic flexibility of the PMVS backbone, the resultant material demonstrates mechanically tunable CPL properties, underscoring its potential as a versatile chiroptical platform for material applications.

## Graphical abstract

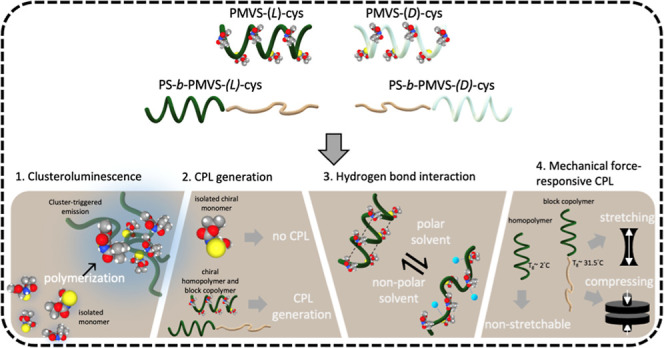

Biomacromolecules perform various physiological functions due to their intricate molecular structures, with chiral molecules, amino acids or carbohydrates. Though the interaction and the organization of the molecules, the secondary to quintenary structure or the helices structure such as proteins and DNA serve as the foundation of the complicated but intriguing biological system. Because helical secondary structures are common in nature (e.g., conch shells), circularly polarized luminescence (CPL)-active materials—excited-state optical properties of chiral systems or self-assembled helices—have attracted great interest for applications in bioimaging, 3D displays, optoelectronic devices, and related fields.^1–5^ Traditional CPL-active materials, comprising π-conjugated molecules,^6^ metal–organic complexes,^7^ and quantum dots,^8^ have shown promise but are hindered by high synthesis costs, biotoxicity, environmental pollution, and aggregation-caused quenching (ACQ).^9^ To address these challenges, clusteroluminescence, based on cluster-triggered emission achieved by the aggregation of nonconjugated and nonaromatic molecules under the action of external forces, has emerged as a viable alternative. This phenomenon is characterized by emissions resulting from through–space interactions of electron-rich heteroatoms (such as O, N, and S) or *n*–π* transitions,^10–12^ thus causing orbital splitting and further luminescence emission. This phenomenon is also commonly found in the natural system, such as sugar or protein.^13^ In particular, Tang et al. have extensively explored these unique luminescence behaviors and mechanisms.^14,15^ attracting growing interest for their potential to overcome the limitations of traditional luminophores.^16–18^ However, so far, very little attention has been paid to CPL.

Since polysiloxane derivatives, including polydimethylsiloxane (PDMS) possess specific properties such as flexibility and transparency, CPL-active materials have been introduced to a polysiloxane matrix and preparation of elastomeric CPL materials was achieved.^19,20^ Although polysiloxane derivatives have been widely employed as matrices for CPL-active systems, the realization of polysiloxane-based materials that can intrinsically produce CPL emission has not yet been achieved. In response to the above challenge, we intend to apply the nontoxic clusteroluminescent material to the polysiloxane by series connection of the chromophore with the rope of linear polysiloxane. The clusteroluminescence could be generated in the concentrated or solid state, and at the same time, the close packing of the chromophore generates the CPL. Because molecular aggregation within polymers is crucial for inducing clusteroluminescence, the design of heteroatom-containing polysiloxane derivatives through covalent incorporation is highly desirable. Recently, we reported a poly­(methyl vinylsiloxane) derivative bearing side chains of enantiomeric cysteine derivatives (PMVS-cys) and demonstrated that PMVS-cys adopts a preferred-handed helical conformation in the film state when cast from nonpolar solvents.^21^ Given that cysteine contains heteroatoms (N, S, and O) and a chiral center, PMVS-cys is expected to exhibit CPL originating from clusteroluminescence.

In this work, we present the first observation of CPL arising from clusteroluminescence in enantiomeric PMVS-cys and its block copolymer, PS-*b*-PMVS-cys systems. Cysteine, a naturally occurring amino acid in the human body, possesses a functional thiol group that readily forms disulfide bonds through oxidation in various enzymes and antibodies. To control the helical conformation of the polysiloxane main chain, both molecular motion and chiral regulation must be carefully managed. The enantiomeric *N*-(*tert*-butoxycarbonyl)-cysteine methyl ester (cys) contains a chiral center, and its bulky *tert*-butyl group provides significant steric hindrance, promoting the formation of a preferred-handed helical conformation in PMVS-cys. The poly­(methyl vinylsiloxane) (PMVS) backbone was synthesized via ring-opening anionic polymerization, and the cys side chains were subsequently introduced through a thiol–ene reaction.^21^ Moreover, a block copolymer (BCP) of polystyrene and PMVS-cys (PS-*b*-PMVS-cys) was also synthesized. Because of the distinct solubilities of the PS and PMVS-cys blocks, the aggregation state of the copolymer can be readily controlled by a selective solvent. Compared with homopolymers, the higher glass transition temperature (Tg) variation in the BCP allows for tunable mechanical properties by adjusting its volume fraction. Chiral induction within the siloxane backbone of polysiloxanes has rarely been reported to date. The development of optically active helical polysiloxanes thus represents a promising avenue for future applications in the biomaterial and biomedical fields (Scheme 1).

**Scheme 1:**
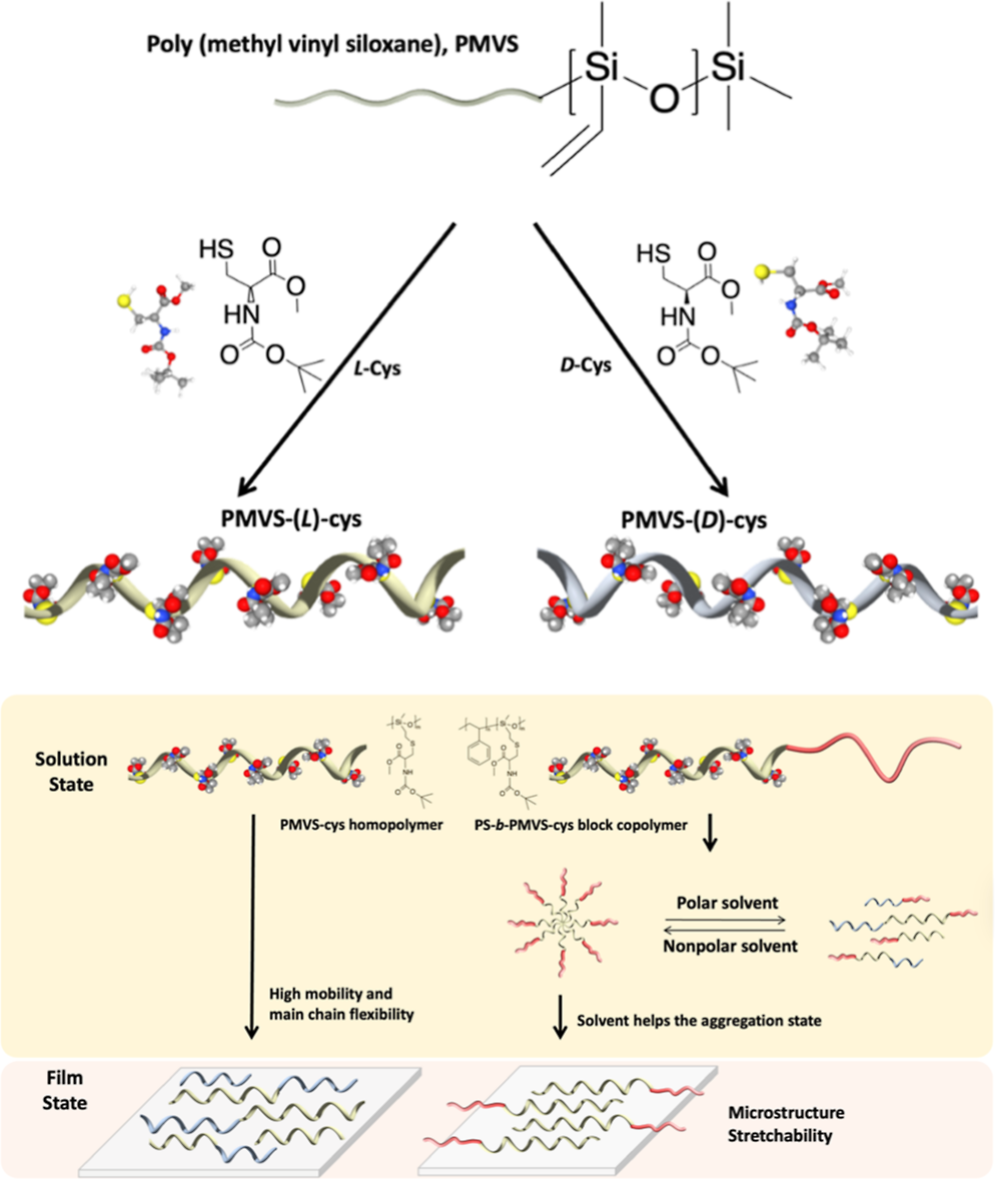
Schematic Illustration of Enantiomeric PMVS-Cys Synthesized by thiol–ene Reaction and Comparison of the Homopolymer and Block Copolymer

To understand the aggregation behavior of the PMVS-cys in Figure 1A, the critical micelle concentration (CMC) of PMVS-cys in chloroform was determined using light scattering measurements at an excitation wavelength of 340 nm. CMC is approximately 5 mM of the cys moieties on the PMVS backbone (∼0.1 wt % PMVS-(l)-cys). Above the CMC, polymer chains tend to aggregate, forming micelle-like clusters or entangled assemblies. Below the CMC, the polymer chains exist primarily in a dispersed, unassociated form, commonly referred to as the single-chain state. As shown in Figure 1B, a clear blue emission can be observed and corresponding fluorescence intensity of PMVS-(l)-cys exhibits a sharp increase around the CMC, suggesting that luminescent clusters form during polymer aggregation. This enhanced blue emission is likely due to the aggregation of the polymer, resulting in the intramolecular or intermolecular through–space interactions necessary for emission (see below in details). Furthermore, compared to free cysteine at the same concentration, PMVS-(l)-cys displays significantly stronger fluorescence, as shown in Figure 1C. This enhancement is attributed to the covalent incorporation of cysteine into the polymer backbone, which facilitates the spatial confinement and proximity of cysteine moieties on the polymer chains. Both the fluorescence behavior of the covalently bound chromophores onto the polymer backbone and the aggregation-caused luminescence enhancement have provided strong evidence for the formation of emissive clusters driven by moieties and even polymer aggregations.

**Figure 1:**
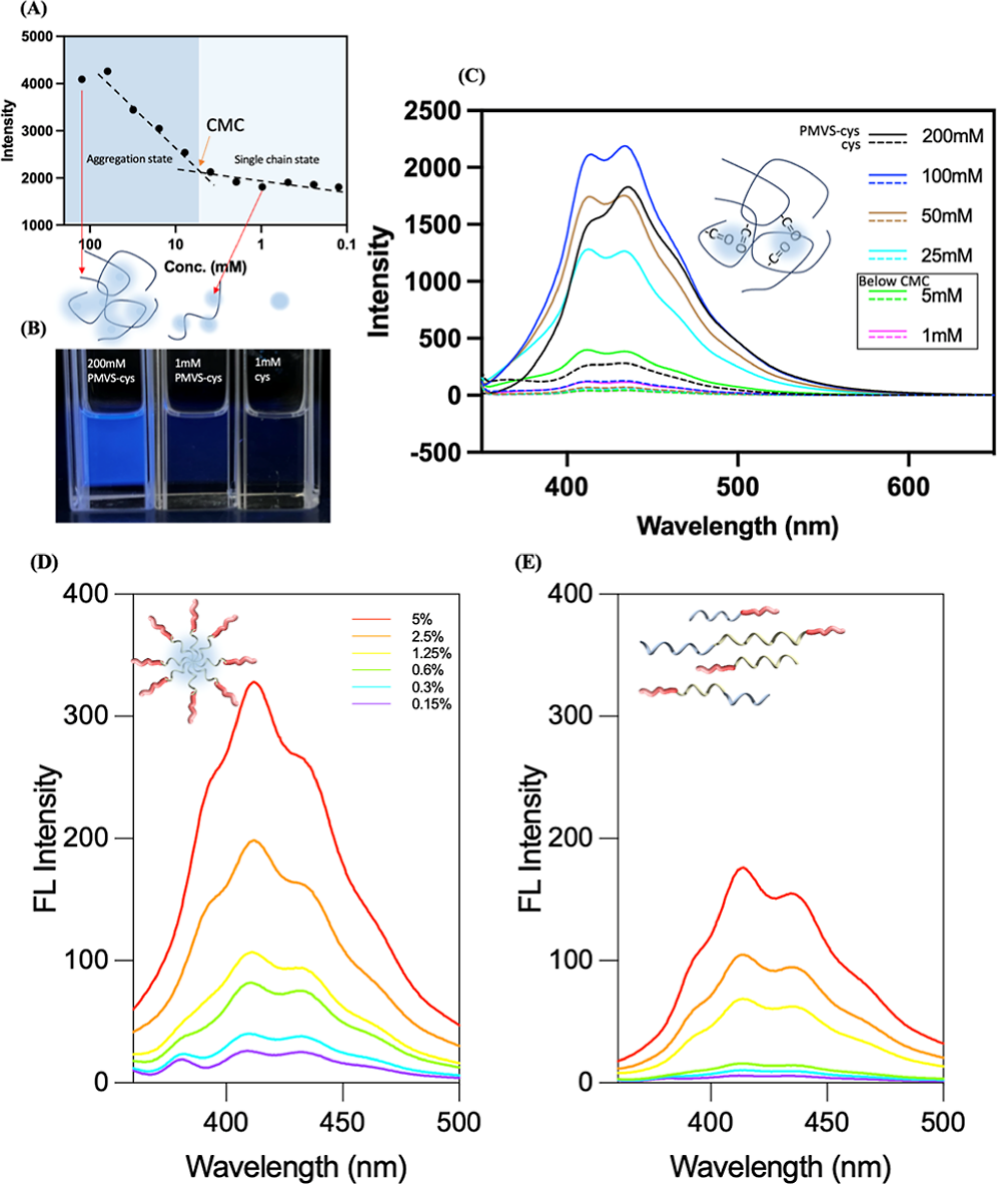
(A) Determination of the critical micelle concentration (CMC) of PMVS-(l)-cys by light scattering at 340 nm excitation. The concentration of PMVS-(l)-cys is calculated by the cys moieties, (B) the picture under 365 nm excitation of 200 mM PMVS-(l)-cys, 1 mM PMVS-(l)-cys, and 1 mM free (l)-cys, and (C) fluorescence intensity of PMVS-(l)-cys and free (l)-cys as a function of concentration under 340 nm excitation. The concentration-dependent fluorescent spectra of PS-*b*-PMVS-(d)-cys in toluene (D) and chloroform (E) in the same concentration level.

To further investigate the clusteroluminescence phenomenon, a block copolymer (BCP) was also synthesized by introducing a polystyrene (PS) segment, yielding a PS-*b*-PMVS-cys. As we know, chloroform serves as a good solvent for both the PS and PMVS-cys blocks, allowing the formation of a homogeneous solution. In contrast, toluene acts as a selective solvent that preferentially solubilizes the PS block, while the PMVS-cys block has poor solubility in toluene. Under these conditions, the polymer self-assembles into micelle-like structures, where the PMVS-cys block forms the core through aggregation, and the PS block forms the solvated corona (Figures S1 and S2). This aggregation behavior enhances through–space interactions among the cysteine moieties in the core, thereby promoting clusteroluminescence. As shown in Figure 1D,E, the fluorescence intensity of PS-*b*-PMVS-(d)-cys in toluene is significantly higher than that in chloroform at the same concentration, supporting the hypothesis that micelle-induced aggregation of PMVS-cys blocks amplifies the emission signal via clusteroluminescence. Also, the CMC of the block copolymer was determined to be approximately 0.6 wt % (Figure S3A). This value is notably higher than that of the PMVS-cys homopolymer (0.1 wt %), attributable to the enhanced solubility of the PS block in chloroform. As shown in Figure S3B,C, both the absorption at 300 nm and the fluorescence intensity at 414 nm increase sharply at the CMC, indicating that aggregation of the cysteine moieties contributes to the observed fluorescence. These results further confirm the clusteroluminescence behavior of the PMVS-cys segment within the block copolymer system.

The chiral transfer of enantiomeric cys to the polysiloxane backbone has been confirmed by vibrational circular dichroism (VCD) in prior studies.^21^ The Si–O–Si stretching shows the split-type Cotton effect, indicating that the enantiomeric cys transfer the helical conformation through the covalent bond (Figure S4). However, the VCD spectra of the two enantiomers are not perfect mirror image, owing to pronounced macroscopic anisotropies in the condensed states. To compare the helical structure in the polymer induced by the cys monomer, we also investigated the (l)-cys monomer at different concentrations by electronic circular dichroism (ECD) spectroscopy. As shown in Figure 2A, the red shift in absorption is also shown in the (d)-cys monomer, which indicates the *J*-aggregation in the chloroform solution. However, the ECD signal always shows a positive state followed by an increasing concentration, which suggests that the contribution of the ECD signal comes from configurational chirality of the (d)-cys monomer, instead of helical conformation. Also, the Cotton effect of the neat enantiomeric cysteine is shown in Figure 2B. Furthermore, the helical conformations of PMVS-cys and PS-*b*-PMVS-cys were further investigated using the ECD spectroscopy. As shown in Figure 2C,D, PMVS-(d)-cys and PS-*b*-PMVS-(d)-cys were measured at varying concentrations in chloroform at room temperature. Notably, PMVS-(d)-cys exhibited a red shift in its absorption maximum from approximately 240 to 250 nm, which was also shown in its ECD signal with increasing the concentration increased (Figure 2C). According to the exciton chirality theory,^22^ this shift and split-type ECD behavior suggest the formation of *J*-type aggregates of the cysteine moieties arranged in a helical manner. In the case of PS-*b*-PMVS-(d)-cys (Figure 2D), a stronger overall absorption was observed compared to PMVS-(d)-cys, due to the higher molar absorption coefficient (ε) of the styrene units. Because polystyrene itself is achiral block, the observed ECD signal originates exclusively from the PMVS-cys block, despite the overlapping absorption bands of the PS and PMVS-cys blocks. Interestingly, a distinct negative ECD band appears near 280 nm at concentrations above 1.25%, indicating the induction of a helical conformation at higher polymer densities. These results suggest that concentration plays a critical role in promoting the helicity of PMVS-cys and PS-*b*-PMVS-cys, with higher concentrations facilitating the stabilization of the helical structure in solution.

**Figure 2:**
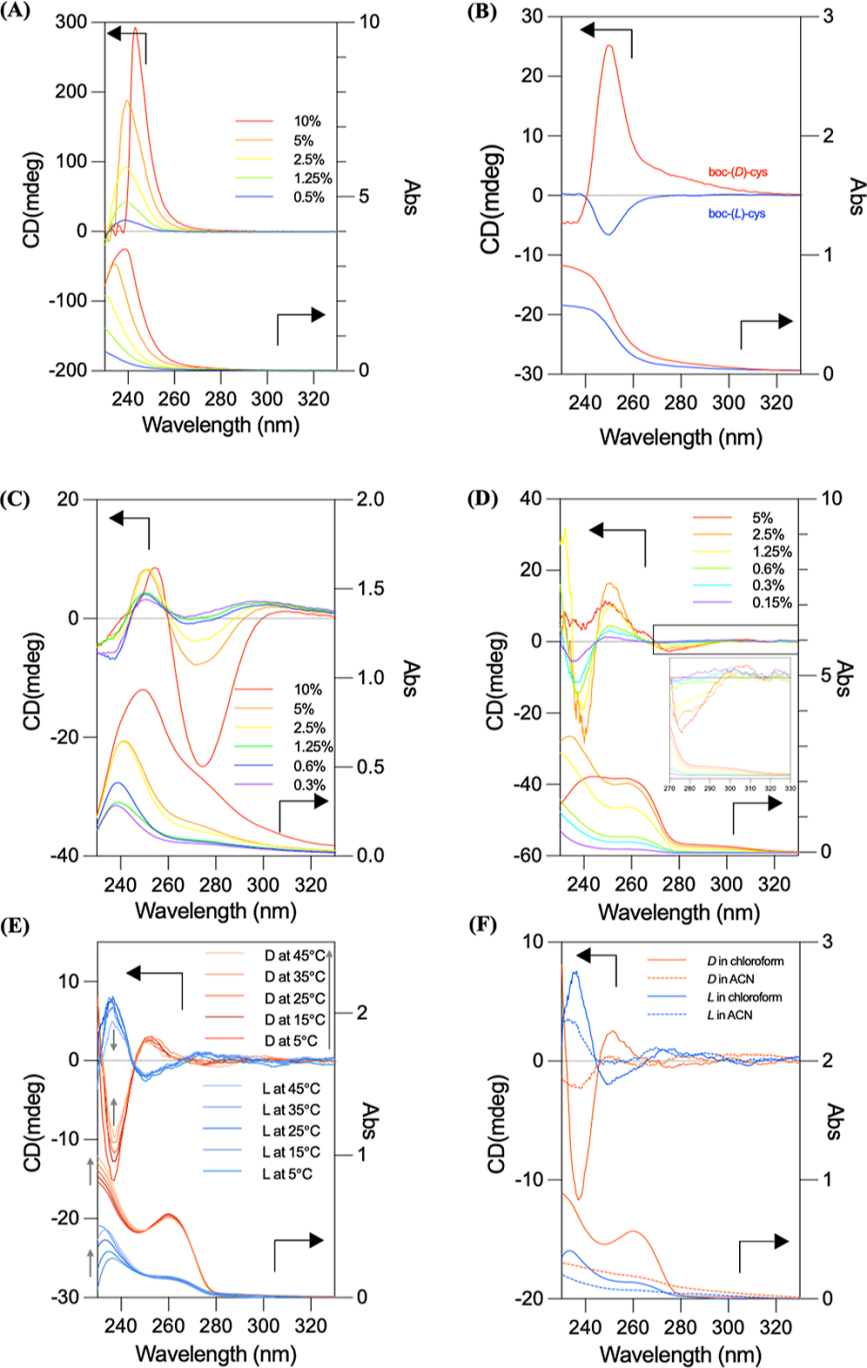
(A) ECD spectra of (d)-cys in chloroform at room temperature over a concentration range of 0.5–10%, (B) ECD spectra of enantiomeric cys monomer at room temperature, (C) ECD spectra of PMVS-(d)-cys in chloroform at room temperature over a concentration range of 0.3–10%, (D) ECD spectra of PS-*b*-PMVS-(d)-cys in chloroform at room temperature over a concentration range of 0.15–5%, (E) ECD spectra of enantiomeric PS-*b*-PMVS-cys in chloroform (0.4%) measured across temperatures ranging from 5 to 45 °C, and (F) ECD spectra of enantiomeric PS-*b*-PMVS-cys (0.4%) at room temperature in chloroform and acetonitrile (ACN).

To further investigate the molecular interactions that govern the helical conformation of this system, the ECD behavior of enantiomeric PS-*b*-PMVS-cys was examined at a range of temperatures and in different solvent polarities. As shown in Figure 2E, a distinct split-type Cotton effect is observed in the enantiomeric PS-*b*-PMVS-cys solution in chloroform, spanning 230–280 nm. Upon heating from 5 to 45 °C, the intensity of the ECD signal at approximately 240 nm gradually decreases, whereas the bands at 260 and 280 nm remain relatively unchanged. Also, the ECD signal is dramatically reduced in acetonitrile (ACN) (Figure 2F) (especially at wavelengths up to 240 nm), indicating that a random coil composition replaced the helices. These results suggest that the helical conformation in PS-*b*-PMVS-cys is primarily stabilized by hydrogen bonding between the cys moieties, making it susceptible to disruption by polar solvents such as ACN, but relatively stable at temperatures below 45 °C. It is noted that the absorption spectra of the two enantiomers appear differ in Figure 2E. To clarify the origin of the absorption spectral discrepancy, we measured the ECD and corresponding UV–vis absorption spectra in both polar and nonpolar solvents. In polar solvents (e.g., chloroform and acetonitrile), the absorption spectra of the two enantiomers exhibit pronounced differences. In contrast, when the solvent is switched to a nonpolar solvent, cyclohexane, the absorption spectra in cyclohexane are nearly superimposable (Figure S5). These findings indicate that solvent polarity significantly affects the absorption characteristics, most likely by modulating the hydrogen-bonding interactions within the two enantiomeric PS-*b*-PMVS-cys.

We can observe the positive ECD signal for (l)-cys moieties and negative ECD signal in (d)-cys moieties in 240–260 nm, while the cys moieties are grafted to the PMVS main chain either in block copolymer or in homopolymer, the other direction of the ECD signal has been generated at a higher absorption wavelength (260–280 nm) according to the split-type Cotton effect. In PS-*b*-PMVS-(l)-cys, the positive ECD signal in 260–280 nm shows P helicity, which is the preferred right-handed helical conformation. However, the PS-*b*-PMVS-(d)-cys shows a negative ECD signal, which indicates the M helicity of the left-handed helical conformation (Table S1). The same helical conformation can be observed in the PMVS-(d)-cys. Thus, the ECD signals in 260–280 nm might indicate the helical direction in the system. To conclude the results we discussed, the intensity in the 260–280 nm range can be further increased by increasing the concentration, indicating that at high concentrations of the polymer, it can facilitate the helical conformation. Also, the polar solvent ACN can disrupt the helical conformation by disrupting molecular interactions.

The helical conformation of PS-*b*-PMVS-cys is primarily stabilized by intramolecular hydrogen bonding between the cys moieties, as confirmed by ^1^H NMR spectroscopy. As shown in Figure 3A, the proton signals assigned to positions *i* and *k* shift downfield in d_8_-toluene compared to their positions in CDCl_3_, indicating deshielding caused by hydrogen bond formation (Figure 3B). This shift suggests that the relatively nonpolar toluene environment promotes stronger hydrogen bonding interactions compared with CDCl_3_. Upon heating the toluene solution to 60 °C, these proton signals shift back upfield, implying the dissociation of hydrogen bonds due to thermal agitation (Figure 3C). In summary, ^1^H NMR analyses reveal that the nonpolar solvent toluene promotes hydrogen bond formation, while heating disrupts these interactions, a trend that is consistent with the behavior observed in polar solvents during CD measurements. Together, hydrogen bonding plays a pivotal role in mediating molecular interactions and stabilizing the helical conformation of PS-*b*-PMVS-cys. Furthermore, Nuclear Overhauser Effect spectroscopy (NOESY) was performed to investigate the spatial proximity of protons and, to probe the presence of hydrogen bonding within the polymer. In NOESY, cross-peaks reveal spatial correlations between nuclei, providing insights into the molecular architecture. Focusing on the proton *k* attached to the nitrogen, which has cross-peaks with other protons or not, because of its involvement in hydrogen bonding with the adjacent carbonyl group, distinct differences were observed across solvents.

**Figure 3:**
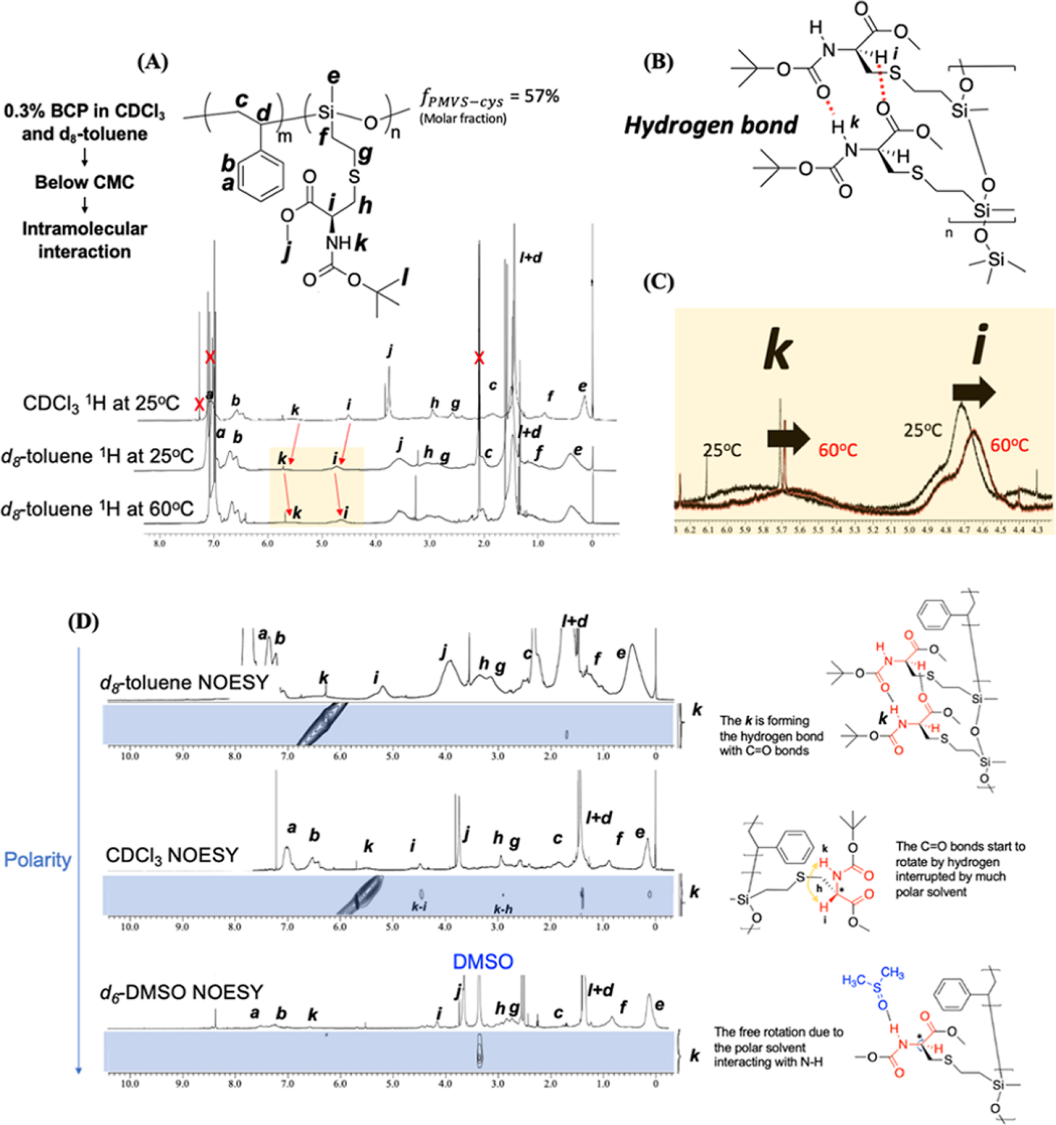
(A) ^1^H NMR spectra of PS-*b*-PMVS-(l)-cys in CDCl_3_ and d_8_-toluene at room temperature and 60 °C, (B) schematic illustration of hydrogen bond formation at proton positions *i* and *k*, and (C) expanded view in NMR signals of the PS-*b*-PMVS-(l)-cys in d_8_-toluene at 25 and 60 °C. (D) NOESY spectra of PS-*b*-PMVS-(l)-cys in d_8_-toluene, CDCl_3_, and DMSO-d_6_, and the chemical structure of the hydrogen bond interaction.

As shown in Figure 3D, the NOESY spectrum in d_8_-toluene does not show cross-peaks between this proton and other nearby protons, suggesting that it is engaged in a hydrogen bond with the carbonyl group, thereby promoting a stabilized helical conformation. In contrast, the NOESY in CDCl_3_ exhibits multiple cross-peaks with surrounding protons, indicative of a more flexible and disordered structure for PS-*b*-PMVS-cys in this moderately polar environment. Moreover, the NOESY in DMSO-*d*_6_ reveals a dominant cross-peak between the protons and the solvent, suggesting that the polar solvent disrupts intramolecular hydrogen bonding. Collectively, these NOESY results align with the observations in ECD spectra, ^1^H NMR, and NOESY spectroscopic results, confirming that hydrogen bonding is th critical interaction stabilizing the helical conformation of PS-*b*-PMVS-cys. This interaction is enhanced in nonpolar solvents, weakened or interrupted by polar solvents, and can be reversibly disrupted by thermal agitation.

Circularly polarized luminescence (CPL) is the phenomenon in which a luminescent material emits light with a defined handedness, either left- or right-circularly polarized. Although conventional spectroscopy captures the ground-state chirality through absorption, CPL allows the investigation of optical activity in the excited state, providing deeper insight into the chiral organization and dynamics of molecular systems. In this study, CPL was examined in PMVS-cys and PS-*b*-PMVS-cys polymers, combining the chirality of cysteine with the cluster-induced luminescence of the nonconjugated polysiloxane backbone. Upon excitation at 340 nm, both PMVS-cys and its block copolymer, PS-*b*-PMVS-cys exhibit CPL activity. As shown in Figure 4A, a positive CPL emission is observed for PMVS-(l)-cys, whereas a negative CPL emission is obtained for PMVS-(d)-cys, while there is no measurable CPL activity for Cys monomers (Figure 4B). These results indicate that the polymeric backbone promotes clustering of the cysteine moieties, not only intensifying the luminescence but also providing a well-ordered chiral self-assembled environment favorable for CPL generation, even though a nonuniform, micelle-like morphologies. Similar CPL behavior is observed in the PS-*b*-PMVS-cys film (Figure 4C). In particular, the higher glass transition temperature (Tg ∼ 33 °C) of PS-*b*-PMVS-cys, compared to PMVS-cys (∼2 °C) (Figure S7), imparts a mechanical character similar to a rubber at room temperature. Mechanical manipulations, including compression and stretching, further reveal the interplay between polymer alignment and CPL response. As shown in Figure 4D, both the bulk samples of PS-*b*-PMVS-(d)-cys and PS-*b*-PMVS-(l)-cys exhibit enhanced CPL intensities after compression, despite yielding the same direction of CPL signs in both of samples. In stretching experiments (Figure 4E,F), polarized light microscopy (PLM) confirms polymer chain alignment, and CPL measurement captures a reversal in the CPL signal between the transparent (unstretched) and blue-tinted (stretched) regions under PLM observations. Moreover, by contrast to enantiomeric cys monomer, the g_lum_ of enantiomeric PMVS-cys homopolymers is higher than the one of PS-*b*-PMVS-cys block copolymers. Furthermore, the g_lum_ can be significantly enhanced after compression (Figure S13). These results demonstrate that the CPL behavior of PS-*b*-PMVS-cys is dictated not only by the conformational chirality but is also highly sensitive to mechanical deformation, highlighting the critical role of polymer chain flexibility and supramolecular ordering in regulating its chiroptical properties. One possible underlying mechanism is the retardation from linear birefringence, which can be amplified under thick sample conditions. As shown in Figures S7 and S8, the sample thickness significantly influences the ECD spectrum around 300 nm for both the block copolymer and the homopolymer. This effect may explain why the thick samples (Figure 4D,E) exhibit a reversed CPL signal.

**Figure 4:**
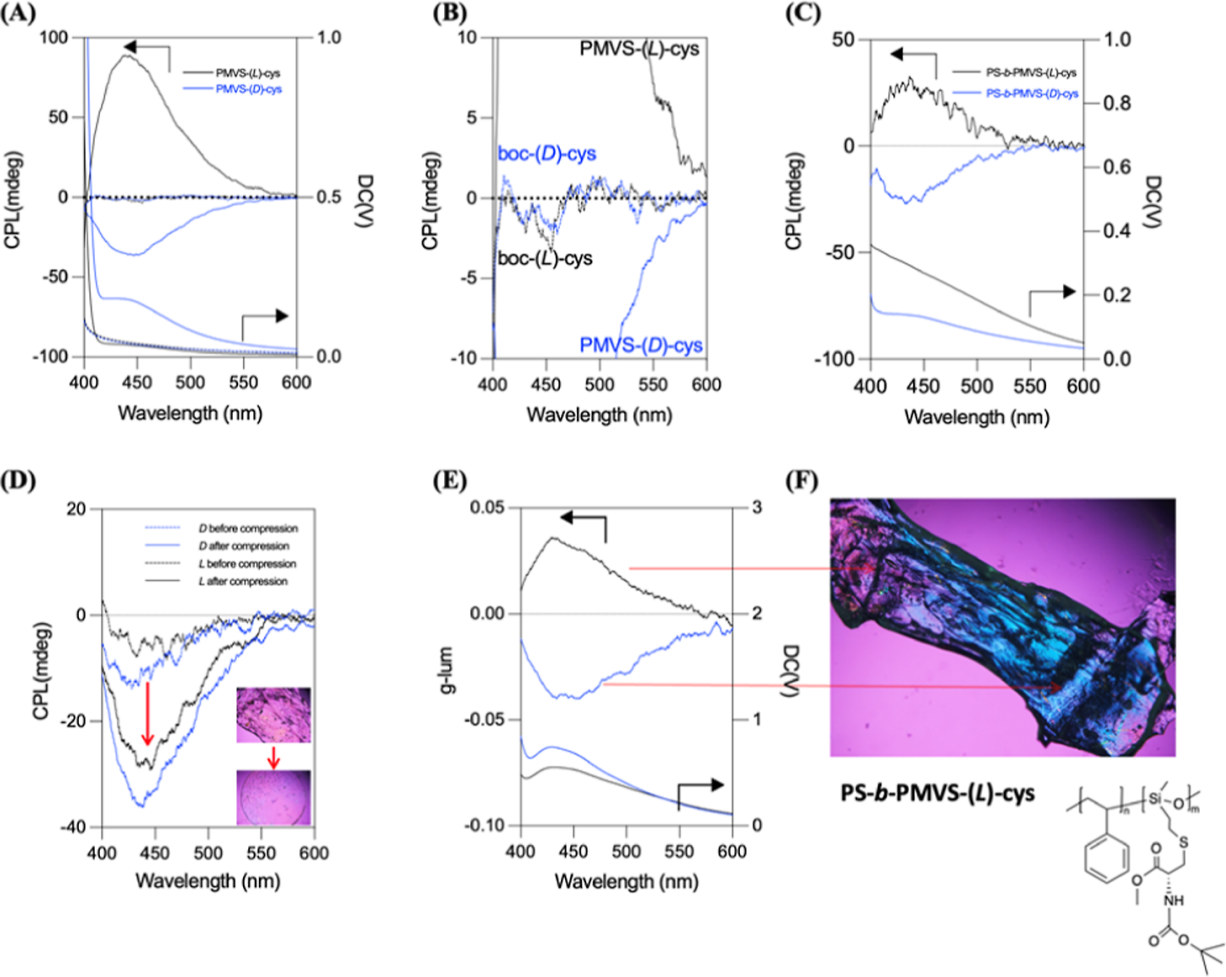
(A) CPL spectra of enantiomeric PMVS-cys and (B) free cysteine, (C) CPL spectrum of PS-*b*-PMVS-cys film, (D) CPL intensities of PS-*b*-PMVS-(d)-cys and PS-*b*-PMVS-(l)-cys bulk samples upon compression, (E) CPL spectra of PS-*b*-PMVS-(l)-cys bulk samples in different parts corresponding to the (F) image of polarized light microscopy.

In conclusion, we have successfully developed a novel class of nonconjugated, nonaromatic circularly polarized luminescent (CPL) polymers based on enantiomeric cysteine-functionalized poly­(methyl vinylsiloxane) (PMVS-cys). The introduction of *N*-(*tert*-butoxycarbonyl)-cysteine methyl ester side chains onto the flexible siloxane backbone enables cluster-triggered emission (CTE) accompanied by distinct chiroptical activity. Spectroscopic analyses at molecular level, including VCD, ECD, and NMR, reveal that the chiral cysteine moieties induce a preferred-handed helical conformation within the polysiloxane chain, which is stabilized predominantly through intramolecular hydrogen bonding. The aggregation of these chiral clusters gives rise to pronounced clusteroluminescence and CPL, with the emission behavior being strongly dependent on solvent polarity, temperature, concentration and chiral self-assembly. Especially, the incorporation of PMVS-cys into a block copolymer (PS-*b*-PMVS-cys) affords a mechanically robust material exhibiting tunable CPL intensity under external stimuli such as compression and stretching. This mechanically modulated CPL originates from supramolecular ordering and polymer chain alignment, demonstrating that the chiroptical properties in such systems can be reversibly tuned by mechanical deformation.

## Supplementary Material



## Notes

Additional experimental details, materials, synthesis, characterization and methods, including photographs of experimental setup (PDF)
