# Supplementary material for: Non-Conjugated Linear Polysiloxane with Cluster-Triggered Circularly Polarized Luminescence
Source: JACS Au. 2026 Jan 13;6(2):1141–7. doi: 10.1021/jacsau.5c01533 (PMC12933315; doi:10.1021/jacsau.5c01533)
Supplement: Supplementary file 1 [file au5c01533_si_001.pdf]

Supporting Information

Non-Conjugated Linear Polysiloxane with Cluster-Triggered Circularly Polarized Luminescence

Hao-Cheng Yu[§], Tomoki Mure[#], Towa Shinoda[#], Chi-Shan Lu[§], Kai Terami[#], Shunsuke Morii[#], Shih-Han Li[‡], Tomoyasu Hirai[#]*, and Ming-Chia Li[‡†]*

[§]*Institute of Molecular Medicine and Bioengineering, National Yang Ming Chiao Tung University, Hsinchu 300, Taiwan*

[†]*Center For Intelligent Drug Systems and Smart Bio-devices (IDS^{2}B), National Yang Ming Chiao Tung University, Hsinchu 300, Taiwan*

[‡]*Department of Biological Science and Technology, National Yang Ming Chiao Tung University, Hsinchu 30010, Taiwan.*

[#]*Department of Applied Chemistry, Osaka Institute of Technology, Osaka, 535-8585, Japan*

Content

1 Materials

1.1 Preparation of Poly (methyl vinyl siloxane) (PMVS)

1.2 Preparation of Enantiomeric PMVS with *(L)*-cysteine Derivatives (PMVS-*(L)*-cys)

1.3 Preparation of Polystyrene-*block*-poly (methyl vinyl siloxane) (PS-*b*-PMVS)

1.4 Preparation of Enantiomeric PS-*b*-PMVS with (*L*)-cysteine Derivatives (PS-*b*-PMVS-(*L*)-Cys)

2 Instruments

3 Supporting Figures

1 Materials

All chemicals were used without future purification except for styrene (FUJIFILM Wako Pure Chemical Corporation, 99%) and 2,4,6-trimethyl 2,4,6-trivinylcyclotrisiloxane (V3) (Tokyo Chemical Industry Co., Ltd., 95%) and tetrahydrofuran (THF) (Merck-Sigma-Aldrich, 99%). Sodium Hydroxide (NaOH, 93%), Magnesium sulfate ($MgSO_4$), Calcium hydride (CaH_2, 95%), naphthalene (98%), hexane (99%), lithium shot, triethylaluminium (1.0M in hexane) and sec-butyllithium (sec-BuLi, 1.3 M in hexane) were purchased from FUJIFILM Wako Pure Chemical Corporation. 2,2-dimethoxy-2-phenylacetophenone (98%), chlorotrimethylsilane (98%) was purchased from Tokyo Chemical Industry Co., Ltd. N-(*tert*-butoxycarbonyl)-*(L)*-cysteine methyl ester (*(L)*-cys, 97%), methanol (MeOH), and hexane (99.0%) were acquired from Merck-Sigma-Aldrich. N-(*tert*-butoxycarbonyl)-*(D)*-cysteine methyl ester (*(D)*-cys, 99%) was purchased from Angene International. Styrene was washed with 15% aqueous solution of NaOH and distilled water, after that styrene was dried with $MgSO_4$. Then styrene was distillated from triethylaluminium just before use. V3 was distillated from CaH_2 just before use. THF was distillated from lithium naphthalene just before use.

1.1 Preparation of Poly (methyl vinyl siloxane) (PMVS)

The reaction was performed under inert gas condition. 30 mL of THF was added to a Schlenk flask with stopcock and cooled to -78°C. sec-BuLi was added to the flask until the color slightly yellow. The flask was placed at room temperature and was kept the temperature until the color change to transparent. Then, the flask was cooled to -78°C again. 0.05 mL of sec-BuLi (0.065 mmol) was added to the flaks and subsequently 2.5 mL (9.7mmol) of V3 was added. The mixture was then removed from cool bath and the reaction was performed at room temperature for 1.5h. The reaction was quenched using chlorotrimethylsilane . The solution was concentrated using an evaporator and subsequently reprecipitated using MeOH.

1.2 Preparation of Enantiomeric PMVS with *(L)*-cysteine Derivatives (PMVS-*(L)*-Cys)

The synthesis has reported in previous paper. 0.10 g of PMVS, 0.38g of *(L)*-Cys, and 0.35 g of 2,2-dimethoxy-2-phenylacetophenone were dissolved in 10mL of THF and the mixture was placed in a flask. Then 365 nm UV-right was exposed for 1 h. The solution was filtered and reprecipitated using hexane. PMVS-*(D)*-Cys was synthesized by placing the *(L)*-Cys to *(D)*-Cys.

1.3 Preparation of Polystyrene-*block*-Poly (methyl vinyl siloxane) (PS-*b*-PMVS)

The reaction was performed under inert gas conditions. 30 mL of THF was added to a Schlenk flask with a stopcock and cooled to -78℃. sec-BuLi was added to the flask until the color turned slightly yellow. The flask was placed at room temperature and was kept at that temperature until the color changed to transparent. Then, the flask was cooled to -78°C again. 0.03 mL of sec-BuLi (0.039 mmol) was added to the flask, and subsequently, 0.7mL (6.0mmol) of styrene was added. The reaction was performed at -78℃ for 30 min and subsequently 0.9 mL (3.5 mmol) of V3 was added at the temperature. Then the flask was placed at room temperature and the reaction was performed at room temperature for 1.5 h. The reaction was quenched using chlorotrimethylsilane. The solution was reprecipitated using MeOH. ^{1}H NMR (400 MHz, CDCl3, δ, ppm): 7.22-6.28(br, C_6H_5, phenyl), 6.28-5.73 (m, C*H*=C*H*$_2$, vinyl), 2.10-1.64 (br, C*H*$_2$-CH, PS main chain), 1.53-1.24 (br, C*H*-Ph, PS main chain), 0.08 (s, C*H*$_3$, main chain). PDI and Mn of PS-*b*-PMVS were 35,000 and 1.4 by SEC (**Figure S9**) and ^{1}H NMR (**Figure S10**), respectively.

In contrast, PMVS-Cys contains a hydrophilic PMVS main chain and hydrophobic cysteine moieties. As a result of this amphiphilic character, SEC analysis using THF as the eluent was not feasible. Based on ^{1}H NMR spectroscopy, the successful introduction of cysteine groups onto the PMVS backbone was clearly confirmed (**Figure S10**). Furthermore, ^{1}H NMR analysis indicated that the molar ratio between the PS and PMVS blocks in PS-*b*-PMVS was 1.0:1.6 (mol%), from which Mn (PS) and Mn (PMVS) were calculated to be 13,500 g·mol^{-1} and 21,500 g·mol^{-1}, respectively. Furthermore, the degree of polymerization (i.e., the number of repeating units) for the PMVS block is calculated to be 250. In the PS-*b*-PMVS-Cys block copolymer, the Mn can be estimated as: Mn (PS-*b*-PMVS-Cys) = 250 × 235.5 + 21,500 + 13,500 ≈ 94,000 g·mol^{-1}.

For the synthesis of the PMVS-Cys homopolymer, we employed PMVS with Mn = 26,000 g·mol^{-1} and PDI (*Đ*) = 1.33. The degree of polymerization of PMVS in this sample is 302. Given the molecular weight of cysteine (235.5 g·mol^{-1}) and the ^{1}H NMR results, the Mn of the PMVS-Cys homopolymer was estimated to be 97,100 g·mol^{-1}.

1.4 Preparation of Enantiomeric PS-*b*-PMVS with (*L*)-cysteine Derivatives (PS-*b*-PMVS-(*L*)-Cys)

0.10 g of PS-PMVS, 0.22 g of boc-*(L)*-Cys, and 0.010 g of 2,2-dimethoxy-2-phenylacetophenone were dissolved in 5 mL of THF in a quartz cell. Then, 365 nm UV light was exposed for 1.5 h The solution was reprecipitated using hexane. PS-*b*-PMVS-*(D)*-Cys was synthesized by placing the *(L)*-Cys to *(D)*-Cys. ^{1}H NMR (400 MHz, CDCl3, δ, ppm): 7.22-6.28(br, C_6H_5, phenyl), 5.45 (br, N*H*), 4.46 (br, C*H*-$COOCH_3$), 3.70 (s, COOC*H*$_3$) 2.87 (br, S-C*H*$_2$-CH-$COOCH_3$), 2.52 (br, S-C*H*$_2$-), 2.10-1.64 (br,

CH_2-CH, PS main chain), 1.53-1.24 (br, CH-Ph, PS main chain), 1.38 (s, C-(CH_3)$_3$), 0.83 (br, Si-CH_2-), 0.08 (s, CH_3, main chain). PMVS-(*D*)-Cys was also prepared using the same method.

2 Instruments

^{1}H(500MHz) nuclear magnetic resonance (NMR) spectra were recorded in $CDCl_3$, d_8-toluene and d_6-DMSO using a JEOL ECZ500R/S1 instrument in both 1D variable temperature and 2D NOESY experiment. Mn and $Đ_M$ were determined by size exclusion chromatography (SEC) using an Agilent 1260 Infinity II instrument (Agilent) with three columns (Tosoh TSKgel Super H2500, TSKgel Super H4000, and TSKgel Super H6000) and 1260 RI detector. THF was used as the eluent with a flow rate of 0.5 mL min−1, and the measurements were performed at 40°C. VCD measurements were performed using VFT4000 (VCD, JASCO). Electronic circular dichroism (ECD) and circular polarized luminescence (CPL) were performed using J-1700 (CD, JASCO) and CPL-300 (JASCO), respectively. Thermogravimetric analysis (TGA) measurement was conducted using PerkinElmer TGA 4000. The measurement was performed at N_2 condition and the heating rate was set to 10°C per min. Differential Scanning Calorimetry (DSC) measurements was performed using NEXTA DSC200 and the heating rate was set to 10°C per min. Transmission electron microscope (TEM) measurements were performed using JEM-2100 operated at accelerating voltage 200kV. The samples measurements were performed without staining. Wide-angle X-ray diffraction (WAXD) and grazing-incidence wide X-ray diffraction (GIWAXD) measurements were performed at TLS 13A1 of National Synchrotron Radiation Research Center (NSRRC) located at Hsinchu, Taiwan. The radiation wavelength of Beamline 13A1 was 1.027 Å and a Mar165 CCD detector was equipped. The distance from the sample to the detector was calibrated with silver behenate and fixed at 178 mm. The incidence angle for GIWAXD measurements was set to 0.12°. The integrated scattering intensity profiles were plotted versus the scattering vector (q), where q was defined as $q = 4\pi \sin(\theta/2)/\lambda$ and θ and λ represent the scattering angle and the wavelength, respectively. Small angle X-ray scattering (SAXS) is measured at TLS 23A of National Synchrotron Radiation Research Center (NSRRC) located at Hsinchu, Taiwan.

3 Supporting Figures

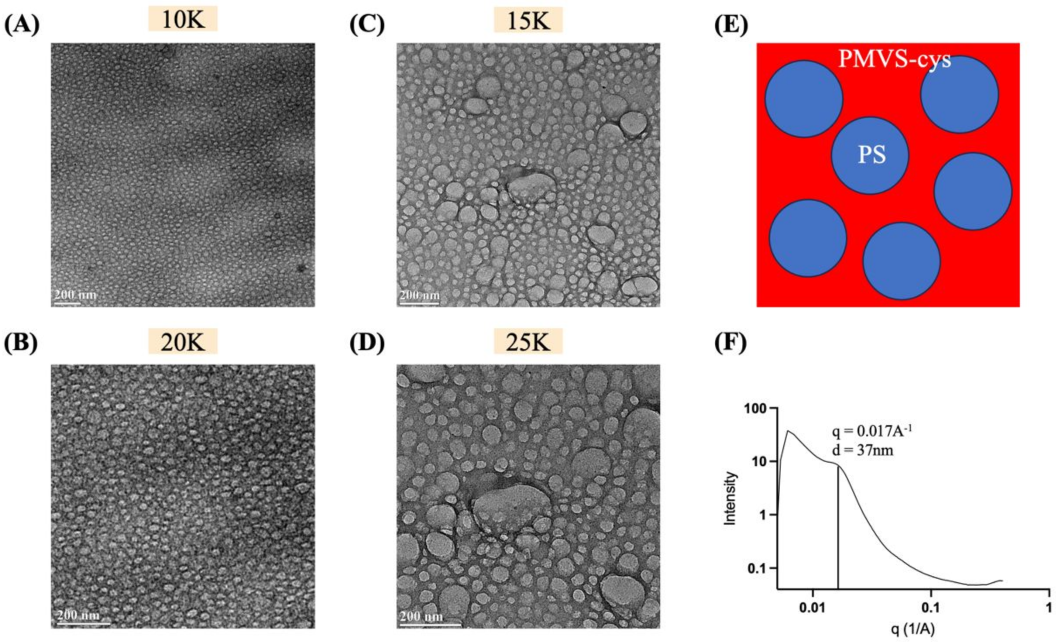

Figure S1. **(A-B)** the TEM picture of PS-*b*-PMVS-*(D)*-cys and **(C-D)** PS-*b*-PMVS-*(L)*-cys in different amplification, **(E)** The illustration of microphase separation (red: PMVS-cys segment; blue: PS segment), and **(F)** corresponding SAXS results of PS-*b*-PMVS-*(L)*-cys.

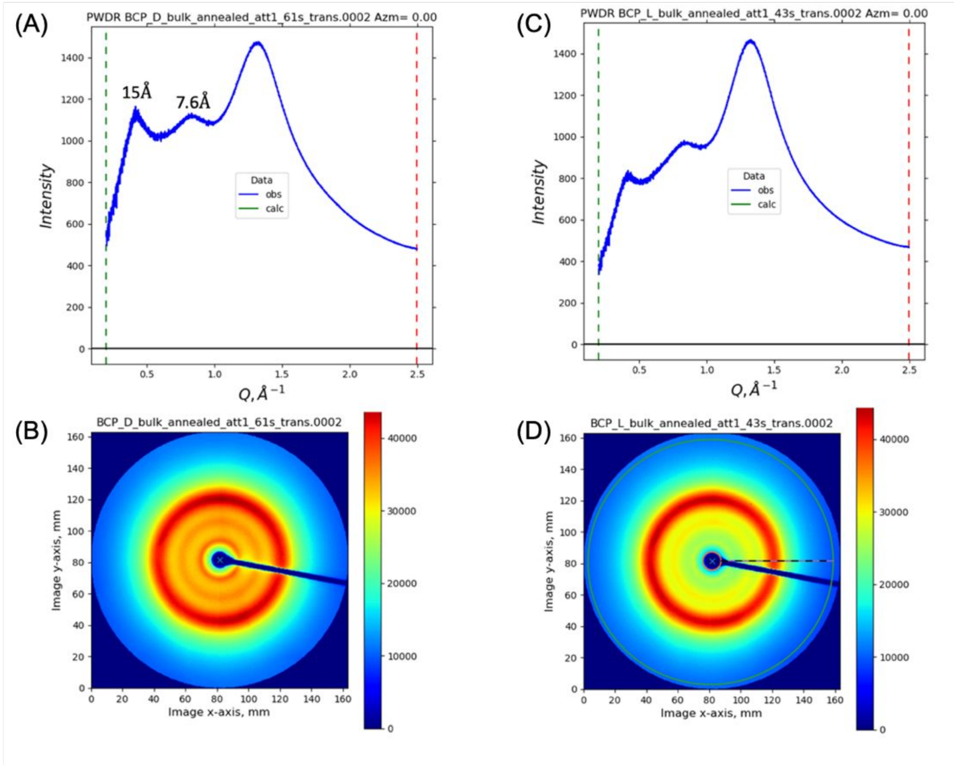

Figure S2. Wide-angle X-ray diffraction (WAXD) analysis of the bulk enantiomeric PS-*b*-PMVS-cys samples: (A) one-dimensional (1D) diffraction profiles and (B) two-dimensional (2D) diffraction pattern of PS-*b*-PMVS-(*D*)-cys, and (C) 1D diffraction profiles and (D) 2D diffraction patterns of PS-*b*-PMVS-(*L*)-cys.

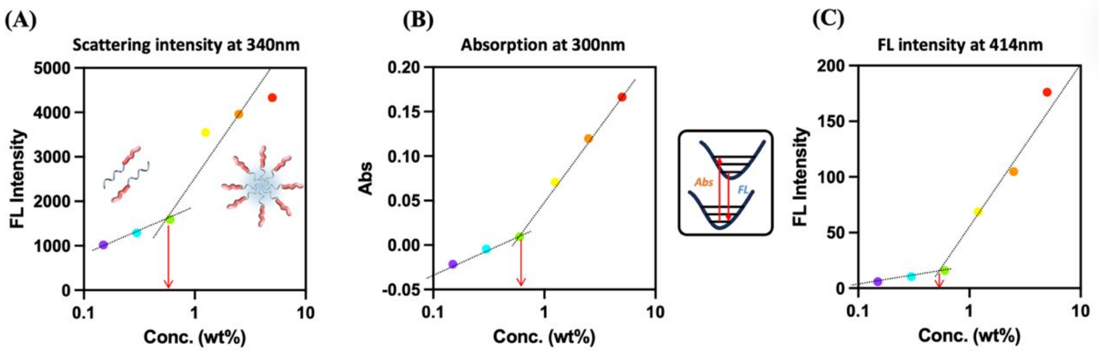

Figure S3. **(A)** the CMC of PS-*b*-PMVS-*(D)*-cys in chloroform, **(B)** the absorption spectrum at 300nm of PS-*b*-PMVS-*(D)*-cys in chloroform, and **(C)** the fluorescent intensity at 414nm of PS-*b*-PMVS-*(D)*-cys in chloroform.

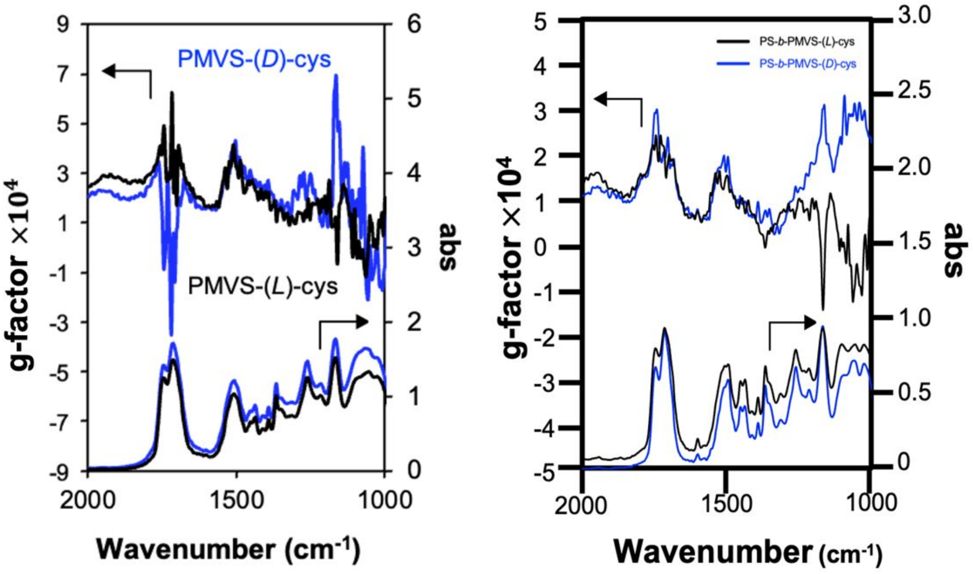

Figure S4. The VCD spectrum of the enantiomeric PMVS-cys (left) and enantiomeric PS-*b*-PMVS-cys (right) measured in the film state.

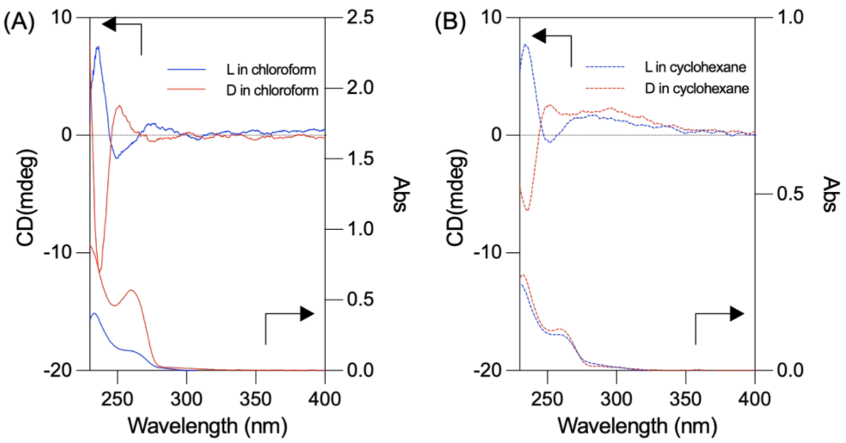

Figure S5. ECD and corresponding UV-vis absorption spectra of enantiomeric PS-*b*-PMVS-cys **(A)** in chloroform (0.4%), and **(B)** in cyclohexane (0.4%) at room temperature.

With respect to the characterization of the ECD signals in **Figure 2**, the absorption dissymmetry factor (g-factor) of the ECD spectra (i.e., g_{abs}) is provided in **Figure S6**. Specifically, the g_{abs} values for PS-*b*-PMVS-(*D*)-cys (**Figure S6C**) reveal that, with increasing concentration, a negative ECD band progressively develops around 280 nm (g_{abs} = -3×10^{-4}), while the ECD feature in the 230–260 nm region is markedly attenuated (from g_{abs} = -6.2×10^{-4} to nearly zero). A similar trend is observed for PMVS-(*D*)-cys (**Figure S6B**), where the ECD signal at approximately 280 nm is intensified and the 230–260 nm band concomitantly diminishes. The g_{abs} value of PMVS-(*D*)-cys reaches -2.2×10^{-3}, which is nearly double that of the (*D*)-cys monomer (-1.3×10^{-3}).

Collectively, these observations demonstrate that concentration plays a critical role in amplifying the helicity of PMVS-cys and PS-*b*-PMVS-cys, with higher concentrations favoring and stabilizing the helical conformation in solution. Furthermore, the primary ECD maximum shifts from ~250 nm to ~280 nm upon polymerization, indicating that the clustered, helical arrangement of chromophores alters their electronic configuration and associated electronic transitions.

In contrast, upon changing the solvent to the polar solvent acetonitrile (ACN), the ECD signal significantly decreased (**Figure S6F**), and the g_{abs} value at approximately 250 nm decreases from -3.6×10^{-4} for PS-*b*-PMVS-(*L*)-cys and 1.6×10^{-4} for PS-*b*-PMVS-(*D*)-cys to nearly zero (**Figure S6E**). This observation suggests that the helical structures are largely disrupted and replaced by a predominantly random-coil conformation.

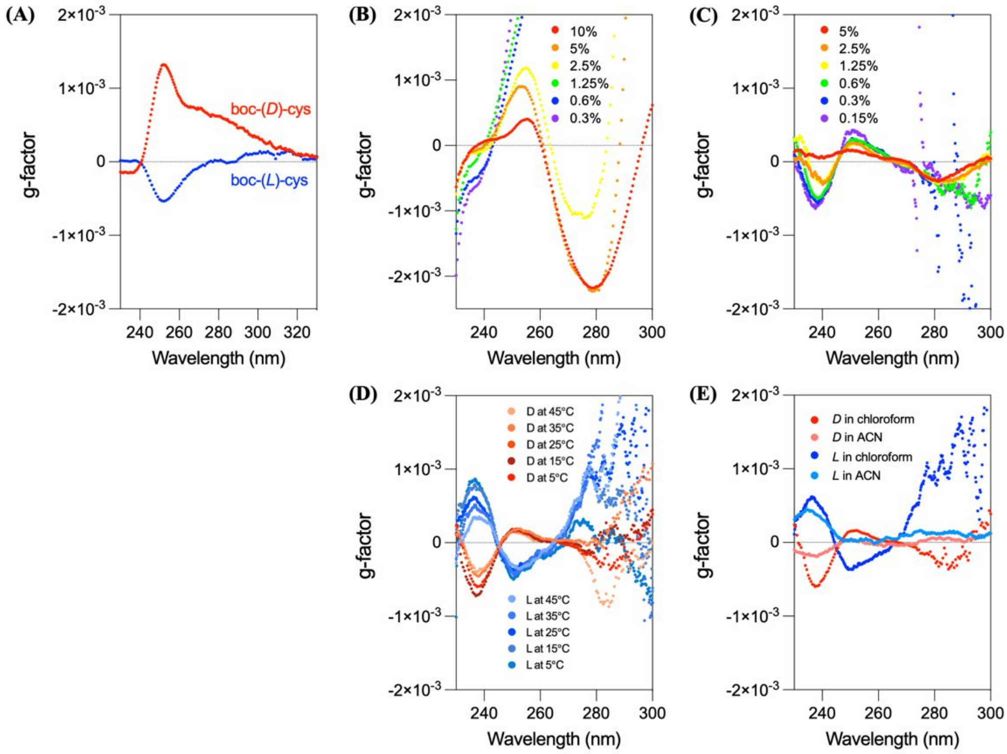

Figure S6. The g-factor (g_{abs}) in the ECD spectra of **(A)** enantiomeric cys monomer at room temperature, **(B)** PMVS-(*D*)-cys in chloroform at room temperature over a concentration range of 0.3–10%, **(C)** PS-*b*-PMVS-*(D)*-cys in chloroform at room temperature over a concentration range of 0.15–5%, **(D)** enantiomeric PS-*b*-PMVS-cys in chloroform (0.4%) measured across temperatures ranging from 5 °C to 45 °C, and **(E)** enantiomeric PS-*b*-PMVS-cys (0.4%) in chloroform and acetonitrile (ACN) at room temperature.

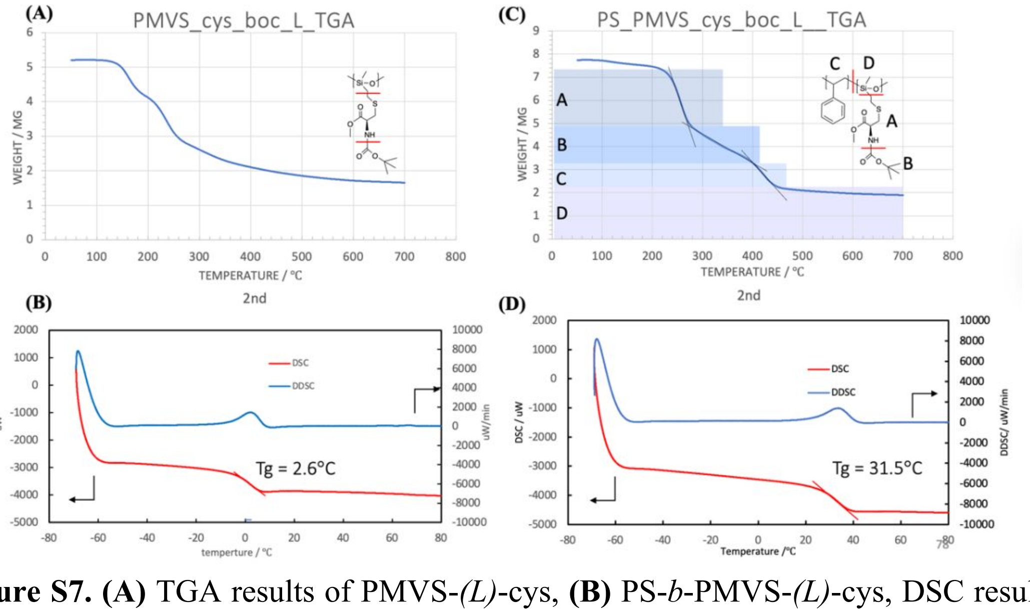

Figure S7. **(A)** TGA results of PMVS-*(L)*-cys, **(B)** PS-*b*-PMVS-*(L)*-cys, DSC results of **(C)** PMVS-*(L)*-cys and **(D)** PS-*b*-PMVS-*(L)*-cys.

Table S1 The recording positive (+) or negative (-) CD signal in enantiomeric cys, block copolymer (PS-*b*-PMVS-cys) and homopolymer (PMVS-cys) at different range of absorption.

	Enantiomer	Wavelength (nm)		
		Below 240	240-260	260-280
Monomer	*L*		-	
	D		+	
Block copolymer	*L*	+	-	+
	D	-	+	-
Homopolymer	*D*	-	+	-

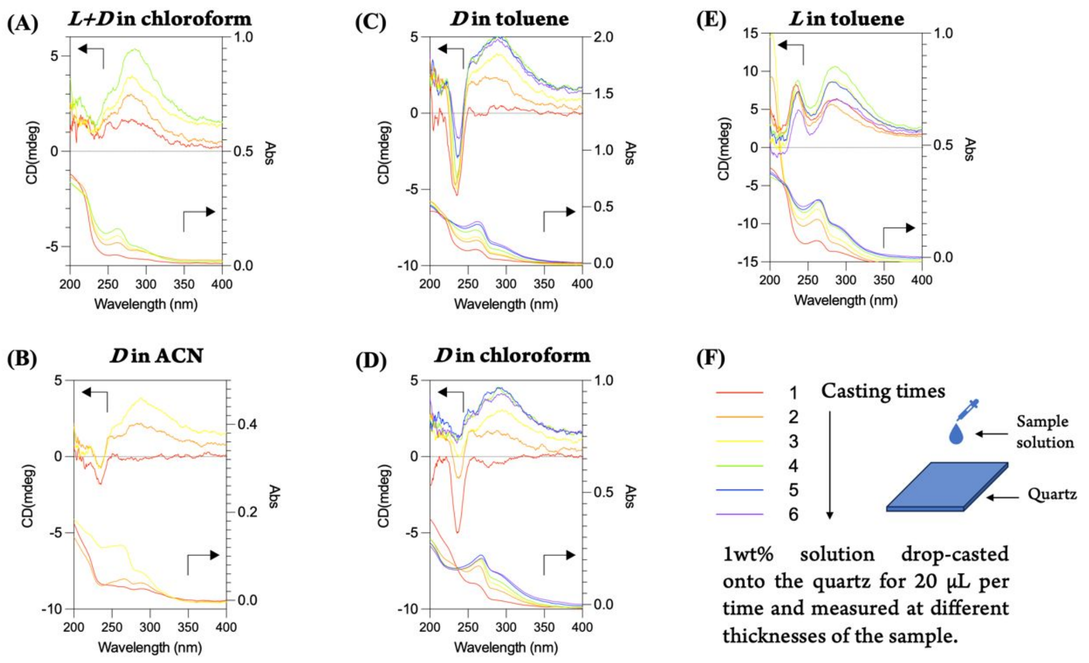

Figure S8. (A) The film state by drop-cast from chloroform of the ECD measurement of mixture of *(L)* and *(D)*-form of the PS-*b*-PMVS-cys, **(B)** from acetonitrile (ACN) of PS-*b*-PMVS-*(D)*-cys, **(C)** from toluene of PS-*b*-PMVS-*(D)*-cys, **(D)** from chloroform of PS-*b*-PMVS-*(D)*-cys, **(E)** from toluene of PS-*b*-PMVS-*(L)*-cys**,** and **(F)** the illustration of casting method.

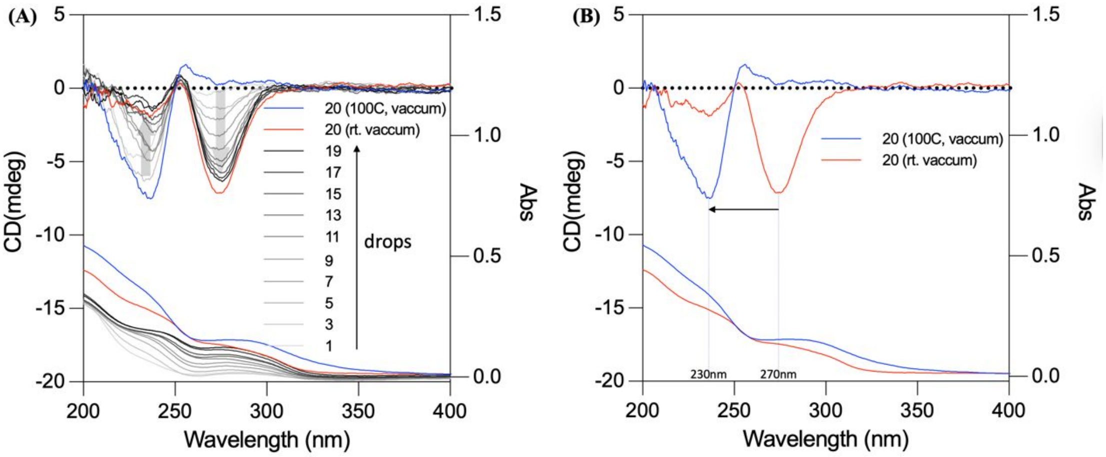

Figure S9. (A) The film state by drop-cast from chloroform of the ECD measurement of homopolymer of PMVS-*(D)*-cys and (B) followed by the heat treatment at 100 °C.

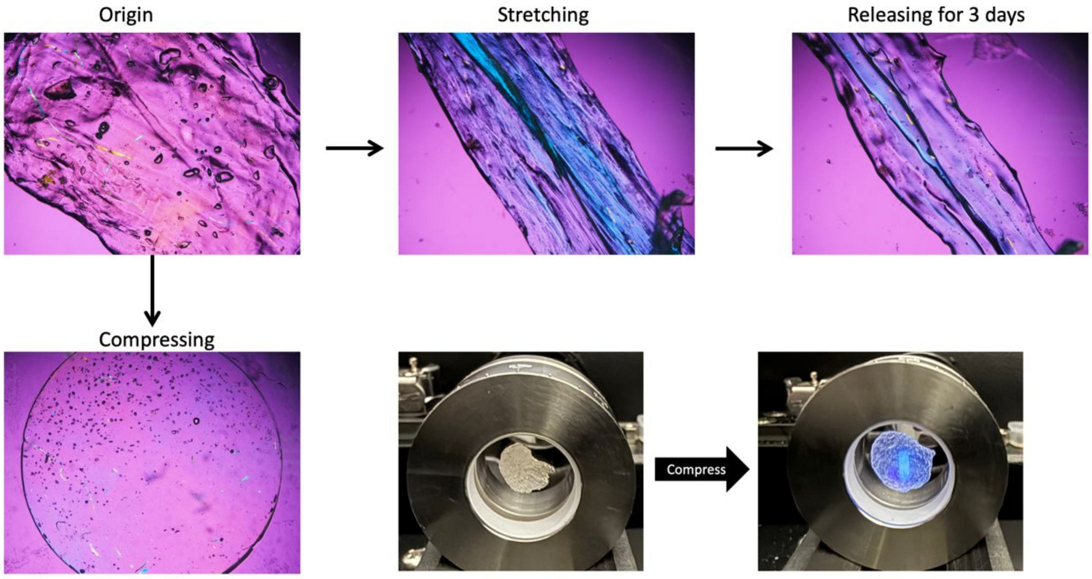

Figure S10. The PLM picture of the mechanical forces applied in the PS-*b*-PMVS-*(L)*-cys, including stretching and compression.

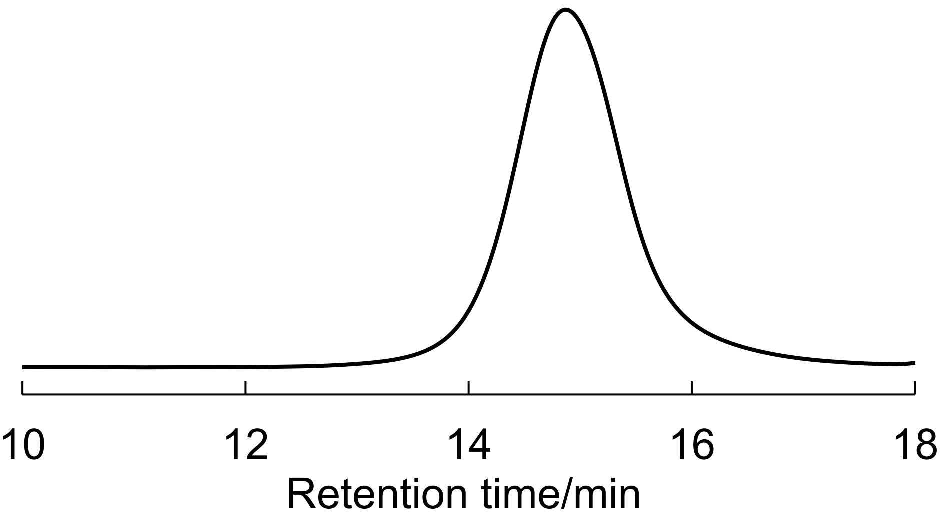

Figure S11. SEC curve of PS-*b*-PMVS.

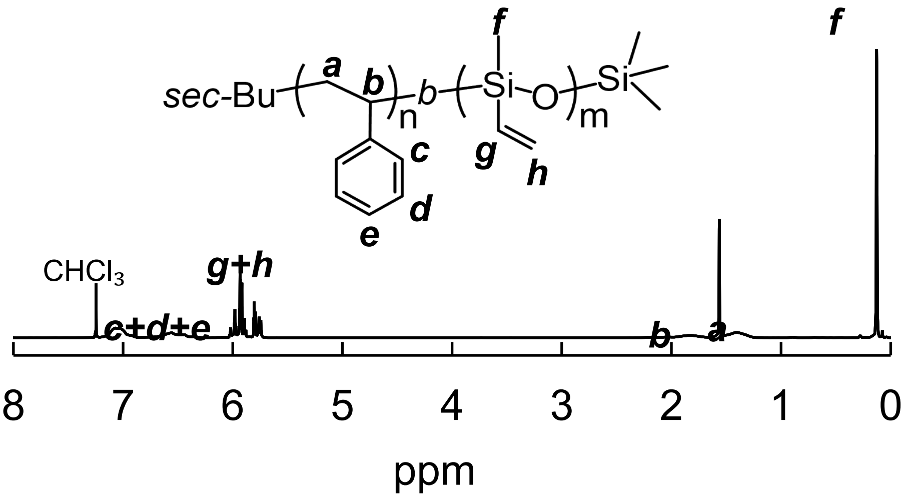

Figure S12. ^{1}H NMR spectrum of PS-*b*-PMVS.

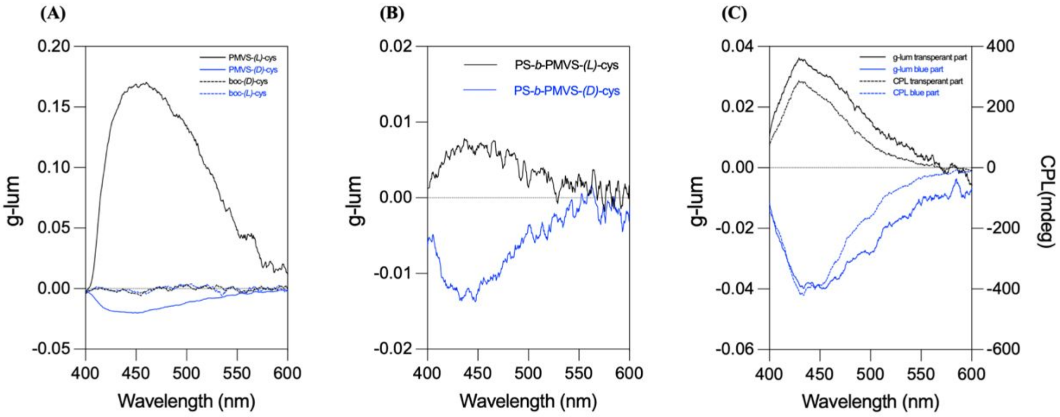

Figure S13. CPL asymmetric factor (g_{lum}) and intensity under different experimental conditions (A) the g-lum of the enantiomeric PMVS-cys and free cysteine, **(B)** the g-lum of the enantiomeric PS-*b*-PMVS-cys film, **(C)** CPL spectra and the g-lum of PS-*b*-PMVS-*(L)*-cys bulk samples in transparent and blue parts after stretching.

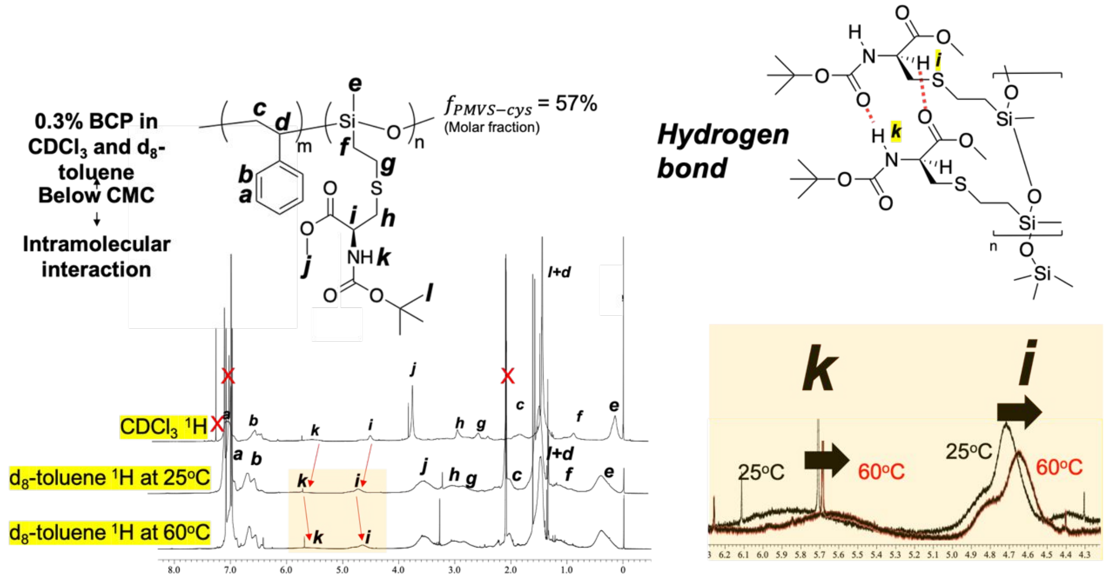

Figure S14. ^{1}H NMR spectra of PS-*b*-PMVS-*(L)*-cys in $CDCl_3$ and *d₈*-toluene at room temperature and 60 °C, and schematic illustration of hydrogen bond formation at proton positions *i* and *k*, and expanded view in NMR signals of the PS-*b*-PMVS-*(L)*-cys in d_8-toluene at 25 °C and 60 °C.

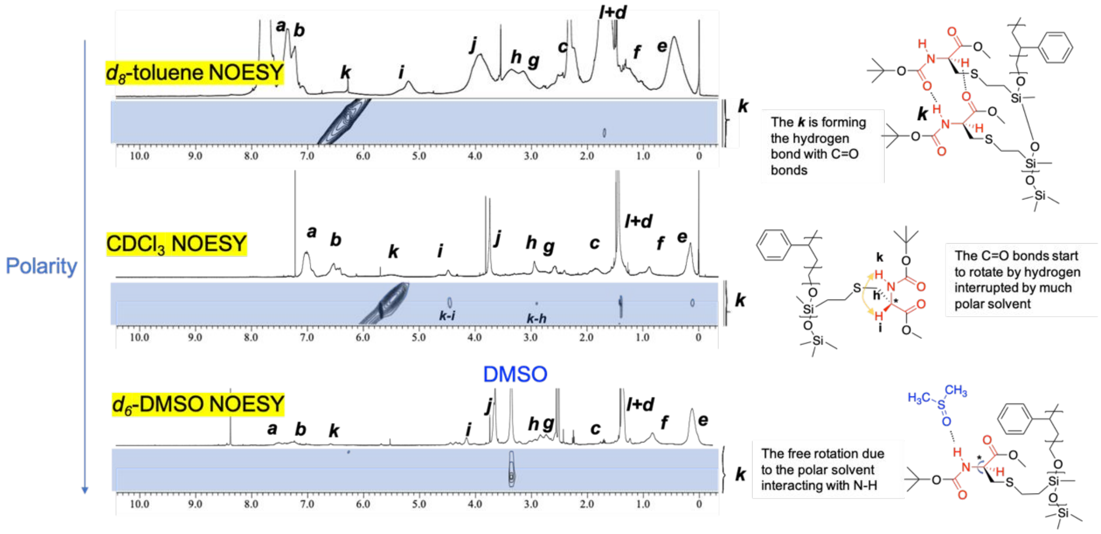

Figure S15. NOESY spectra of PS-*b*-PMVS-*(L)*-cys in d_8-toluene, $CDCl_3$, and d_6-DMSO, and the chemical structure of the hydrogen bond interaction.

Reference

1. Tomoki Mure, Yakumo Kinoshita, Hinari Sakai, Shunsuke Morii, Hsin-Ni Wu, Tsz-Ming Yung, Hao-Cheng Yu, Kodai Nagashima, Wataru Higashiguchi, Noboru Ohta, Teruaki Hayakawa, Yoshinobu Nakamura, Syuji Fujii, Ming-Chia Li, and Tomoyasu Hirai, *ACS Macro Letters* **2024** *13* (5), 537-541
